# Identification of Specific Pathogen-Infected sRNA-Mediated Interactions between Turnip Yellows Virus and *Arabidopsis thaliana*

**DOI:** 10.3390/cimb45010016

**Published:** 2022-12-30

**Authors:** Ruiyang Yu, Xinghuo Ye, Chenghua Zhang, Hailong Hu, Yanlei Kang, Zhong Li

**Affiliations:** School of Information Engineering, Huzhou University, Huzhou 313000, China

**Keywords:** differential expression, functional sRNA, machine learning, cross-kingdom, TuYV

## Abstract

Virus infestation can seriously harm the host plant’s growth and development. Turnip yellows virus (TuYV) infestation of host plants can cause symptoms, such as yellowing and curling of leaves and root chlorosis. However, the regulatory mechanisms by which TuYV affects host growth and development are unclear. Hence, it is essential to mine small RNA (sRNA) and explore the regulation of sRNAs on plant hosts for disease control. In this study, we analyzed high-throughput data before and after TuYV infestation in Arabidopsis using combined genetics, statistics, and machine learning to identify 108 specifically expressed and critical functional sRNAs after TuYV infection. First, comparing the expression levels of sRNAs before and after infestation, 508 specific sRNAs were significantly up-regulated in Arabidopsis after infestation. In addition, the results show that AI models, including SVM, RF, XGBoost, and CNN using two-dimensional convolution, have robust classification features at the sequence level, with a prediction accuracy of about 96.8%. A comparison of specific sRNAs with genome sequences revealed that 247 matched precisely with the TuYV genome sequence but not with the Arabidopsis genome, suggesting that TuYV viruses may be their source. The 247 sRNAs predicted target genes and enrichment analysis, which identified 206 Arabidopsis genes involved in nine biological processes and three KEGG pathways associated with plant growth and viral stress tolerance, corresponding to 108 sRNAs. These findings provide a reference for studying sRNA-mediated interactions in pathogen infection and are essential for establishing a vital resource of regulation network for the virus infecting plants and deepening the understanding of TuYV virus infection patterns. However, further validation of these sRNAs is needed to gain a new understanding.

## 1. Introduction

*Arabidopsis thaliana* serves as a key model organism in the plant kingdom. Turnip yellows virus, formerly known as Beet western yellows virus (BWYV) [1], affects plants with deformed yellowing leaves and stunted plant growth [2,3]. It is widespread worldwide and has a wide host range, including more than 100 plants from 21 families. It significantly negatively impacts local economies by drastically reducing oilseed rape yields in Europe and Australia [4,5]. Recent tests have discovered the virus in Turkey’s peanuts and the UK’s peas [6,7].

A sophisticated innate immune system, similar to the non-viral pathogenic system and broadly separated into the pathogen-associated molecular pattern (PAMP)-triggered immunity and effect-triggered immunity protects plants from viral infections [8]. Furthermore, in addition to the molecular level described above, the mechanism of TuYV infestation in Arabidopsis may also act through sRNA. MicroRNA (miRNA) and small interfering RNA (siRNA), which together can trigger RNA interference (RNAi), are two types of sRNA. Double-stranded RNA (dsRNA) is converted into single-stranded RNA (ssRNA) during this process by RNase type III enzymes known as Dicers. One of the two sRNAs then forms RISCs (RNA-induced silencing complexes), and these RISCs invariably contain Argonaute (Ago) protein to cleave target RNAs [9,10], which inhibit protein synthesis [11]. DCL2 generates 22-nucleotide sRNAs and plays a prominent role in antiviral RNA silencing [12], while virus-derived small RNAs can also target host transcripts. RNA silencing offers a regulatory network through which many immune systems can fine-tune their responses. The function of sRNAs in immunity extends far beyond a straightforward antiviral defensive system [13]. A recent study shows that the generation of vsiRNA loaded into AGO protein in CMV infection of Arabidopsis can mediate the silencing of endogenous genes, which may be a side effect of anti-virus [14]. Since CMV and TuYV are both plant RNA viruses, we can speculate that TuYV infection of Arabidopsis may also have similar anti-virus side effects.

Machine learning is vital for understanding plant virus pathogenesis and host-virus interactions. Machine learning allows accurate and rapid analysis of high-throughput data from virus-infected plants to identify gene regulatory networks (GRNs) and novel host factors involved in host-virus interactions [15]. Few studies currently use machine learning methods on plant data compared to animals and humans. It is also difficult to directly identify functional non-coding RNAs in a predictive manner in cross-species studies due to a lack of data and poor understanding [16]. We are mining functional sRNAs in this study to provide a small idea. The idea is to evaluate the sRNA produced in TuYV-infected *Arabidopsis thaliana* through machine learning technology to verify its sequence specificity.

Current studies on the regulation of TuYV infestation have focused on the molecular level. The interactions with the host are still not fully understood, e.g., Marion Clavel et al. revealed the complexity of antiviral RNA silencing of phloem-restricted polerovirus TuYV and prompted a re-evaluation of the role of its inhibitors in silencing P0 during actual infection [17]. Furthermore, the regulatory role of sRNAs in the interaction of TuYV with Arabidopsis remains poorly understood. This study used high-throughput data and artificial intelligence models, including SVM, XGBoost, and CNN, to assess the sRNAs produced during the TuYV infestation of Arabidopsis. The data set was created by separating several ranges and multiples, and machine learning models were utilized for prediction to validate their specificity better. We performed genomic matching to infer their origin, followed by target gene prediction and functional enrichment analysis to identify these sRNAs that may be involved in regulating growth and defense in Arabidopsis following viral infestation. This study provides a research design to regulate TuYV-infected plants, constructing specific sRNA datasets and validating them using machine learning. The potential functional sRNAs obtained may be of great value for the mechanism of TuYV virus infestation against plants, which may also provide a new outlook for the regulation of plants by other viruses and virus control.

## 2. Results

### 2.1. Assessment of the Specificity of sRNA before and after Infection

The results showed that SVM and CNN worked best and that sRNA expression levels before and after infection, if 10 times different, already had an apparent specificity. We selected several machine learning techniques for medium abundance testing.

**Figure 1:**
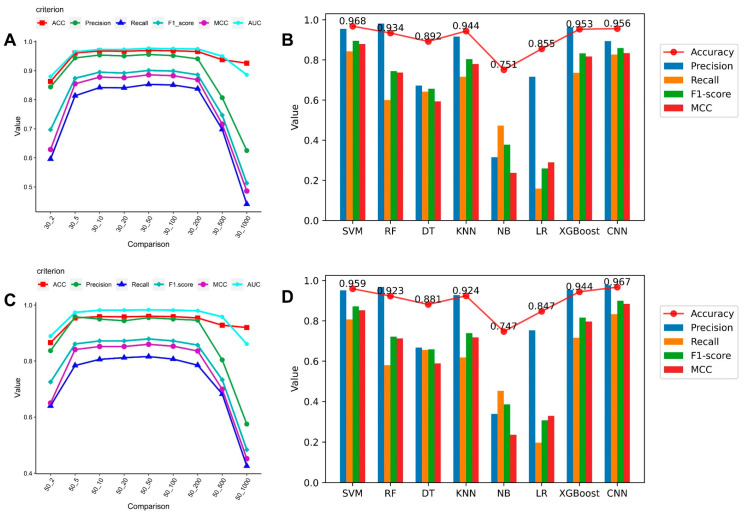
Line graph and histogram of sRNA sequence specificity verification using machine learning: (**A**) The SVM performance trend at x times change in the 30 RPM value data set. (**B**) The evaluation result of each machine learning model at the 10 times change in 30 RPM value. (**C**) The SVM performance trend at x times changes in the 50 RPM value data set. (**D**) The evaluation result of each machine learning model at the 10 times change in 50 RPM value.

Processing features using the SVM is easier and quicker, and we were able to calculate the evaluation scores for the variance of the SVM at each fold using the medium abundance (see Figure 1A,C). Our results show that this trend inflects 10 times before and after infection, with each indicator higher. The ratios of positive and negative samples were 866:3677 and 508:2377 for samples with a 10-fold change in 30 RPM value and a 10-fold change in 50 RPM value, respectively. Evaluation using multiple machine learning models on these two datasets of medium abundance RPM values yielded promising results (see Figure 1B,D for the evaluation results). For SVM at the 10-fold change in 30 RPM value and CNN at the 10-fold change in 50 RPM value, respectively, the maximum accuracy was 0.968 and 0.967. We added the Matthews correlation coefficient (MCC) assessment since the ratio of positive to negative sRNA samples screened against actual data was uneven and close to 1:4. The MCC of our created CNN can reach 0.884 on stricter filtering conditions and fewer training sets. In this experiment, considering the small sample size and the good results, we believe that a large number of sequence-specific sRNAs are more likely in the case of a 10-fold change in the 50 RPM value, and we used sRNAs under that range for further studies. We confirmed the chosen sRNAs from the standpoint of sequence specificity by combining the analysis of the aforementioned several machine learning models. The results show that both SVM and CNN are more effective with medium abundance RPM value datasets, and SVM is more practical and reliable than other machine learning techniques.

### 2.2. Target Gene Prediction Results

Following target gene prediction, we show partial results in Table 1. The repressed gene AT1G63450, which codes for XYLOGLUCAN-SPECIFIC GALACTURONOSYLTRANSFERASE1, is related to the growth of Arabidopsis root hair tips [18], is predicted to be the target of sRNA ‘GAGGACGAGAACATGAACTGGA’. The GDP-L-galactose phosphorylase encoded by one of the genes anticipated to be targeted for repression by sRNA “AATTGCCGGAAGGGAAACTC”, AT5G55120, may be crucial in the regulation of ascorbic acid production and has an impact on the growth of Arabidopsis [19]. By changing cellulose deposition and cell wall elongation in Arabidopsis, one of the genes anticipated to be targeted for repression by sRNA “AAGACAAGACTCTAAAACTCCT”, AT2G18800, plays a significant role in primary root growth [20]. The sRNA “TACGGATGAGCAAGTGCTGGACT” predicts targeting of AT5G61160, a guanidinyl coumaroyltransferase (AtACT) that catalyzes the final reaction in the biosynthesis of HCAA, a secondary metabolite involved in defense of plants against pathogens. Some researchers have demonstrated that HCAA is responsible for defense against pathogens in Arabidopsis [21]. The gene AT1G61140, also known as embryonic sac development arrest 16 (EDA16), is anticipated to be targeted by the sRNA ‘GTCGAAGAAGGAGGCTTGCCC’ and is related to immunomodulatory pathways [22].

### 2.3. Regulatory Pathways Associated with Growth and Defense in Arabidopsis thaliana as a Result of Enrichment Analysis

**Figure 2:**
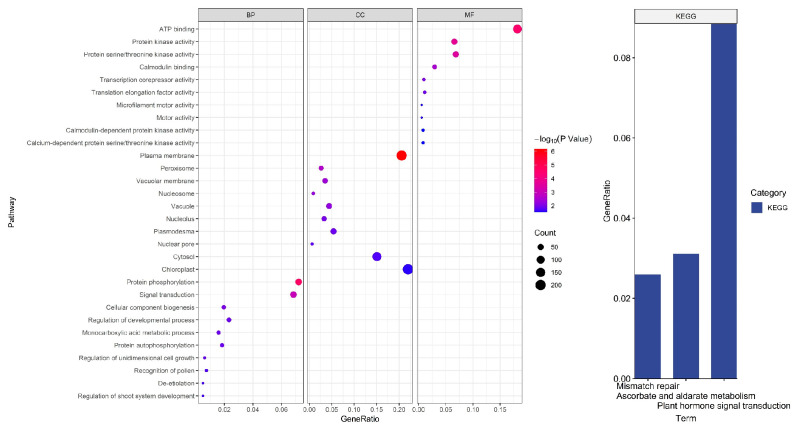
Target genes function enrichment analysis and KEGG pathway results.

The functional enrichment results included 38 biological processes (GO), 18 molecular functions (GO), 22 cellular components (GO), and three KEGG pathways. According to GeneRatio sorting, we primarily mapped the top 10 biological processes (GO) and three KEGG pathways, as shown in Figure 2.

Five biological processes, including protein phosphorylation, protein autophosphorylation, immune system process regulation, regulation of defensive responses, and regulation of defense responses to other organisms, are all related to defense-related regulation.

The regulation of viral gene transcription, viral intracellular/intercellular motility, viral genome replication, viral infectivity of host plants, plant innate immunological response and counter-defense, and viral particle assembly, in particular, is greatly influenced by phosphorylation [23].

Four biological processes regulate growth and development: the regulation of developmental processes, cellular elements in biogenesis, one-dimensional cell growth, and regulation of the root-shoot system’s development.

Two functional protein kinase activities and calmodulin assemblies associated with viral action show enrichment in molecular function. During plant–virus interactions, protein phosphorylation is a frequent post-translational alteration that regularly takes place. Usually, host protein kinases control viral pathogenicity and infectivity by phosphorylating viral proteins [24]. Some researchers demonstrated that in response to the viral invasion, wound-induced calcium signaling activates genes relevant to RNAi [25].

Among cellular components, vesicles are linked to elements involved in defense. Recent studies have demonstrated the relationship between vesicle acidification and plant immunity and the necessity of vesicle acidification for plant antiviral immunity. By interfering with vacuolar acidification, viral proteins prevent autophagic breakdown and advance viral infection [26].

In KEGG analysis, pathways related to defense may involve plant hormone signal transduction. The primary hormones that control a plant’s ability to defend itself against infections are salicylic acid (SA), jasmonic acid (JA), ethylene (Et), and abscisic acid (ABA) [27].

## 3. Discussion

According to the study, pathogenic processes may have developed in Arabidopsis due to TuYV infection. In this study, we noticed that 247 sRNAs might be virus-derived and predicted these 247 sRNAs to target host Arabidopsis genes, of which 108 sRNAs affected Arabidopsis growth and defense. The lack of degradation data halted further study. In order to better understand the 108 sRNAs that may play a vital role in the regulation of Arabidopsis and determine whether they also exist in other plants infected with the TuYV virus, further research requires additional experimental data.

In the realm of virus-infected plants, there are currently no unquestionable and consistent selection criteria for differential sRNAs. For TuYV-infected Arabidopsis differential sRNAs, we chose medium abundance RPM values to limit random drift and get more sRNAs. Discoveries may result from different screening techniques or differential sRNAs acquired at various RPM abundances.

We trained several machine learning models to verify the sequence specificity of sRNAs. However, these models predicted infection only for sRNAs expressed at levels above medium abundance RPM values, even though sRNAs expressed at other abundance values may be critical to the infection process. The method screens out sRNAs based on medium abundance at different folds to generate positive and negative samples and can achieve a prediction accuracy of approximately 96.8%. Most of the machine learning models used in this study performed well on the medium abundance specific sRNA dataset, with the best SVM outcomes coming from samples with a 10-fold change in 30 RPM value and the best CNN outcomes coming from samples with a 10-fold change in 50 RPM value. Multiple models were used to confirm that sRNAs are sequence-specific and may even be functional. Functional sRNAs of TuYV viruses have received minimal study, and more research is required to comprehend this subject thoroughly.

## 4. Materials and Methods

### 4.1. Data Source and Experimental Design

**Figure 3:**
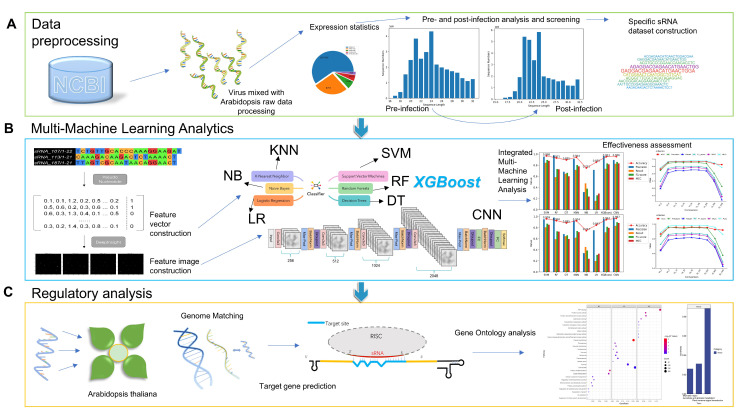
Entire experimental design scheme: (**A**) Data pre-processing stage: Process high-throughput data to count sRNA expression, obtain sRNAs with significant expression changes before and after infection, and divide multiple ranges to construct specific sRNA datasets. (**B**) Multi-machine learning analysis phase: constructing sequence features of the obtained positive and negative samples and analyzing different ranges using multiple models. (**C**) Regulatory analysis stage: We conducted genomic matching of the analyzed sRNAs to obtain possible sources and then started target gene prediction and performed functional enrichment analysis to obtain gene function regulation results in Arabidopsis.

We retrieved TuYV-infested Arabidopsis sRNA HTS data(GSE176378) from GEO (Gene Expression Omnibus; http://www.ncbi.nlm.nih.gov/geo/; accessed on 10 November 2021) [28]. Samples were obtained from Arabidopsis wild plants before infection and 16 days after infection by the TuYV virus. The detailed description can be queried in the experiment of Marion et al. [17]. We downloaded the genomes of the TuYV virus and Arabidopsis from NCBI (https://www.ncbi.nlm; accessed on 15 March 2018). The Arabidopsis Information Resource (TAIR; http://www.arabidopsis.org/; accessed on 11 July 2019) [29] was used to collect Arabidopsis cDNA sequences. We show the entire experimental design scheme in Figure 3.

### 4.2. Data Pre-Processing

The expression levels of each sRNA before and after infection, or the total number of sequences in the data file, are necessary to identify sRNAs with appreciable changes in expression. Researchers often use read count and read per million (RPM) values to assess sRNA expression. RPM values were employed in the statistics to account for the slight variance in sRNA length and to remove the sequencing depth bias. Normalized counting of an sRNA = (Original read count of the sequence/Total read count of the data set) × 10^6^ [30]. We removed adapters and low-quality sequences from the raw sequencing data during pre-processing and short sequences (including “N”) that bases cannot recognize. Following the removal of the brief sequences with no reads, the expression values of the sequences were standardized in terms of RPM. Illumina performs adapter attachment during library preparation, and adapter removal is necessary to obtain the right sRNA sequence. Trimmomatic and Cutadapt are the two most popular adapter removal tools currently available. In this work, we deleted the sequenced sequence 3’ segment adapter TGGAATTCTCGGGTGCCAAGGAACTC using the Trimmimatic-0.39 tool [31]. After Trimmimatic removes the adapter from the sRNA sequence, the adapter is no longer present in the FASTQ file.

In the pre-processing stage, a script is used to control the length and count the RPM values to produce a file that only contains sequences and RPM values with a sequence length of 18–25 nt and only one occurrence.

### 4.3. Construction of Specific sRNA Datasets before and after Infection

**Figure 4:**
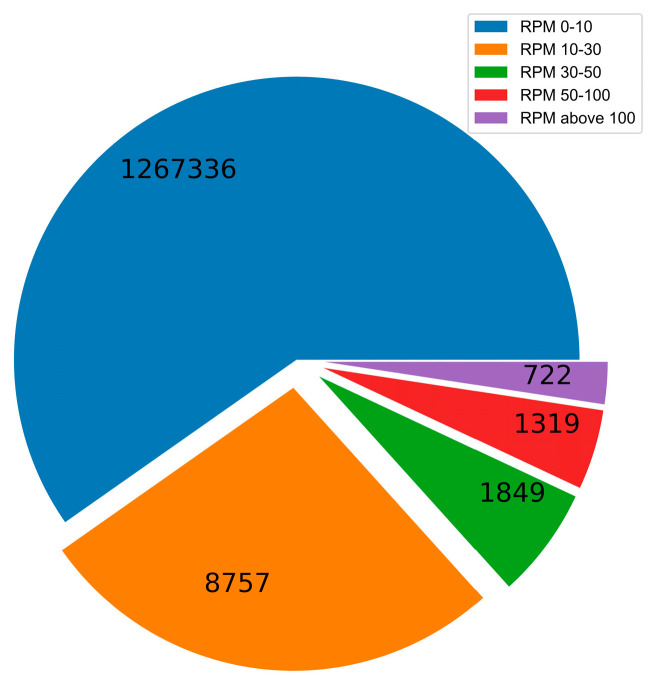
The sequence reads of each RPM value distribution of sRNA.

For constructing specific sRNA datasets before and after infection, random drift and identifying precise expression changes [32] are two challenging difficulties. Low-expression sRNAs may experience random drift due to the infrequent repetitive sequencing during the library construction. The absence of differentially expressed sRNAs may come from overly stringent screening criteria for highly expressed sRNAs. Some researchers classified sRNAs into three groups, namely high abundance (>100 RPM), medium abundance (10–100 RPM), and low abundance (1–10 RPM). Highly and moderately expressed sRNAs are less susceptible to random drift [32]. We show in Figure 4 the distribution of sequence reads and RPM values of sRNA after TuYV infection, where the reads of low abundance RPM values (0–10) were 1267336, the reads of medium abundance RPM values (10–100) were 11925, and the reads of high abundance RPM values (>100) were 722. The possibility of random drift for RPM values less than 10 is relatively high, while >100 is too strict and tends to lose numerous potential sRNAs, so we used medium-abundance RPM values of 30 and 50.

At present, there is no unified standard for qualitative differential expression. Common methods include the log2 ratio, multiple comparisons [33,34,35,36,37]. To determine the difference pre- and post-infection, we use the difference in multiple relationships before and after infection to distinguish the different degrees of its expression. We use the numerous associations of 2 times, 5 times, 10 times, 20 times, 50 times, 100 times, 200 times, 500 times and 1000 times to test.

We conducted data pre-processing to get the sRNA files before and after infection, including sequences and RPM values. The total number of sRNAs present before and after infection was 1,279,983 after statistics.

After script screening, we obtained sRNA samples at each fold change above medium abundance and used the samples above and below each time as positive and negative samples, respectively, with the positive and negative samples’ labels set to 1 and 0. The training and test sets were split up and trained using various machine learning models to ensure that the resulting sRNAs were sequence-specific.

### 4.4. Characteristic Expression Methods for sRNA

We used the pseudo-ribonucleic acid composition features of RNA sequences to generate 104-dimensional feature vectors using the SC-PseDNC-General method of Pse-in-one [38] for input to the machine learning model. We apply DeepInsght [39] to convert these feature vectors into feature images for input to the convolutional neural network. We combine the above feature construction method to propose a new image generation method, which converts sRNA sequences into 2-dimensional (2D) images. We also build convolutional neural networks (CNNs) for the sRNA classification.

The feature vector is normalized to lie between (0, 1) in each machine learning method using maximum-minimum normalization. The formula states the following:

(1) $$y=\frac{x-\min}{\max-\min}$$

where max and min are the feature vector’s maximum and minimum values, y is the normalized version of the feature vector, and x is the original version.

### 4.5. Machine Learning Model Training and Exploration

Machine learning is trained on the parameters using tenfold cross-validation and grid search and implemented in scikit-learn 0.24.2 [40], where the SVM uses a radial basis function (RBF) kernel function to train the model. XGBoost [41] separate implementation. Convolutional layers, pooling layers, batch layers, dropout layers, and fully connected layers are all used in the 16-layer architecture of CNN and implemented in Pytorch 1.9.1 [42]. The input (*N*, *C_in_*, *H*, *W*) and output (*N*, *C_out_*, *H_out_*, *W_out_*) of the convolutional layer can be precisely described as follows:

(2) $$\mathrm{out}\left(N_{i},C_{out_{j}}\right)=bias\left(C_{out_{j}}\right)+\overset{C_{in}-1}{\underset{k=0}{\sum }}weight\left(C_{out_{j}},k\right)⋆input\left(N_{i},k\right)$$

where ⋆ is the valid 2D cross-correlation operator, *N* is the batch size, *C* denotes the number of channels, *H* is the height of input planes in pixels, and *W* is the width in pixels.

We explored multiple classification models with the following parameters:

- Support vector machine (SVM; RBF): regularization parameter (2.91), kernel (RBF), kernel coefficient (0.91);
- RandomForest (RF): number of trees (100), the minimum number of samples for splitting a node (2), minimum number of samples at a leaf node (1);
- DecisionTree (DT): criterion (gini), minimum number of samples for splitting a node (2), minimum number of samples at a leaf node (1);
- K-Nearest Neighbor (KNN): number of nearest data points (5);
- GaussianNB (NB): portion of the largest variance of all features (1 × 10^9^);
- LogisticRegression (LR): penalty (L2), max iteration (3000);
- XGBoost: booster (gbtree), learning_rate (0.3);
- CNN: Parallel convolution filter widths (all filter widths 3), Convolution dimension (First layer [3256], Second layer [256,512], Third layer [512,1024], Fourth layer [1024,2048]), Pooling layer width (uniformly 2), Pooling layer calculation (uniformly choose the maximum pooling layer), Batch normalization between the convolution and pooling layers, use dropout2D after batch normalization (dropout rate 0.5), Activation function (chosen uniformly among ReLU and Sigmoid), use dropout1D in the last two fully connected layers (Dropout rate 0.5), Learning rate (0.001).

### 4.6. Genome Matching and Target Gene Prediction

After choosing sRNAs and confirming the sequence specificity of those sRNAs using machine learning, we aim to identify their sources and investigate their functions, revealing their regulatory roles through genome matching and predicting them against host target genes.

We used Bowtie [43] for genomic matching after analyzing sRNA in the previous step. Next, target gene prediction was carried out on Arabidopsis cDNAs to identify target genes. In the first step, we use the NCBI data to construct the TuYV genome index library and index package. Set the mismatch option to 0, perform rigorous matching on the process, and output SAM files for all matches. Extract the files with only the reserved sequences by using the script. In the second step, we used the target gene prediction program psRNATarget [44]. Before target gene prediction, the sRNA files were converted to FASTA files using a script, and the script changed the base T in the sRNA sequences to U. Next, we carried out target gene prediction.

### 4.7. Gene Functional Enrichment Analysis

We obtained GO and KEGG biological processes by additional enrichment analysis after using the DAVID database [45]. We selected all KEGG pathways and showed 10 GO pathways according to GeneRatio in descending order of biological process (BP), cellular component (CC), and molecular function (MF).

## 5. Conclusions

This study explored the regulation of TuYV infection in *Arabidopsis thaliana* at the sRNA level. Specific sRNA datasets were constructed by reducing random drift and obtaining more sRNAs, with a recommended RPM value of medium abundance. Multiple machine learning models were combined to validate the sequence-level specificity of sRNAs and achieved approximately 96.8% accuracy, and we assessed SVM and 2D convolutional CNN as demonstrating optimal performance for this study. The 108 functional sRNAs, possibly from the TuYV virus, were targeted and predicted to affect four biological processes of growth and five biological processes of defense in Arabidopsis. Due to the lack of degradation group data, we cannot further verify these sRNAs, but the occurrence of these sRNAs in other plants infected by the TuYV virus is also worth exploring in more experiments. Our highlight is that from the perspective of machine learning, we have verified the sequence specificity of some sRNAs produced by TuYV infecting Arabidopsis, obtained 108 sRNAs, and analyzed the potential role of these sRNAs in Arabidopsis. These sRNAs may not have been taken seriously in previous studies.

## Figures and Tables

**Table 1:** 108 sRNA partial displays: RPM values, counts, length, and target genes before and after sRNA infection. RPM1 and RPM2 are the sRNA expressions before and after infection, Count1 and Count2 are the respective sRNA counts before, and after infection, Len is the length of sRNA, and Target is the predicted target gene.

Seq	RPM1.	RPM2.	Count1.	Count2.	Len.	Target
GAGGACGAGAACATGAACTGGA	1.255	1092.535	35	30662	22	AT1G63450.1, AT1G10590.3, AT1G30790…
CATGGAACTCAATGGCTGTCAT	1.210	719.360	34	20188	22	AT4G37200.1, AT3G51940.1, AT1G47128…
AATTGCCGGAGAAGGGAAACTC	0.360	532.210	10	14934	22	AT5G55120.1, AT2G27810.1, AT2G27810…
AAGACAAGACTCTAAAACTCCT	0.450	469.215	12	13167	22	AT2G18800.1, AT5G58210.3, AT5G58210…
CCGAAGCAGAACTGAAGAGCCT	0.400	360.825	11	10126	22	AT1G24620.1, AT1G50460.2, AT1G50460…
……						
AGCTAGTGGGGGTTCTGACAC	0.000	53.845	0	1511	21	AT5G45455.1, AT5G08390.1, AT2G47490.1
GATGATCGCCATAGCACTTGAT	0.035	52.235	1	1466	22	AT1G70520.1, AT3G51220.1
CGCTACTATCATCGCCGGGGGT	0.100	52.095	3	1462	22	AT5G35604.1, AT3G19430.2, AT4G13730…
TACGGATGAGCAAGTGCTGGACT	0.090	50.830	2	1426	23	AT3G59790.1, AT3G07400.1, AT5G61160.1
GTCGAAGAAGGAGGCTTGCCC	0.000	50.550	0	1418	21	AT1G61140.3, AT4G31210.3, AT4G31210.1…

## Data Availability

Not applicable.
